# Effects of Caprylic Triglyceride on Cognitive Performance and Cerebral Glucose Metabolism in Mild Alzheimer’s Disease: A Single-Case Observation

**DOI:** 10.3389/fnagi.2014.00133

**Published:** 2014-07-16

**Authors:** Brian Andrew Farah

**Affiliations:** ^1^High Point Regional Division of University of North Carolina Health Care, High Point, NC, USA

**Keywords:** Alzheimer’s disease, ketone bodies, glucose, apolipoprotein E, Montreal cognitive assessment, mini mental state exam

## Abstract

**Objective:** To examine the effect of 109 days of caprylic triglyceride (CT) in a 70-year-old male with mild Alzheimer’s disease (AD).

**Background:** Cerebral metabolism is limited to glucose under most conditions, and diminished cerebral glucose metabolism is a characteristic feature of AD. Another substrate available for cerebral metabolism is ketone bodies. Ketone bodies (KB) are normally derived from fat stores under conditions of low glucose availability as an alternative energy substrate to glucose. KB can also be produced by oral administration of CT. Prior studies suggest that the alternative energy source of CT may improve cognitive function due to mild to moderate AD, by circumventing the diminished glucose metabolism.

**Method:** The effect of CT was analyzed in a single-case of mild AD with cognitive alterations in an open label study. Study outcomes included the Montreal cognitive assessment (MoCA), mini mental state exam (MMSE), and 18-fluorodeoxyglucose (18F) positron emission tomography (FDG PET) scans.

**Results:** After 109 days of CT, MoCA scores changed from a baseline value of 24–28, and MMSE scores changed from 23 to 28. No changes were observed on FDG PET scans.

**Conclusion:** The results suggest that, in a case of mild AD, CT may have affected cognitive function, assessed by means of MMSE and MoCA, although glucose uptake and metabolism remained unchanged.

## Introduction

### Therapeutic rationale

Alzheimer’s disease (AD) is progressive, neurodegenerative disease characterized by a decline in cognitive abilities. The pathological hallmarks of AD include accumulation of senile plaques and neurofibrillary tangles in the brain. Variants in three genes, the two presenilin genes (*PSEN1/2*) and the amyloid precursor protein (*APP*) are known to cause early-onset forms of AD. Amino acid changes encoded by the gene variants in the *APP* and *PSEN1/2* genes ultimately lead to the accumulation of amyloid beta and phosphorylated tau and the insidious early-onset of the disease. These findings, combined with the pathology, strongly implicate dysregulation in the processing of the APP protein as a central player in the disease (Schellenberg and Montine, 2012). However, the majority of patients with AD do not carry disease causing variants in *APP*, *PSEN1*, or *PSEN2*. Instead, the major risk factors for sporadic AD are increasing age and the possession of the epsilon 4 (E4) variant of the apolipoprotein E (*APOE*) gene (Padilla and Isaacson, 2011).

The brain is one of the most metabolically active organs in the body, and under most conditions, relies almost exclusively on glucose for its energy needs. Using 18F-2-deoxy-2-fluoro-d-glucose (18FDG), a positron emitting tracer, the cerebral metabolic rate of glucose (CMRglc) can be measured. 18-Fluorodeoxyglucose (18F) positron emission tomography (FDG PET) studies in the early 1980s compared AD subjects with normal controls and found significant diminished cerebral glucose metabolism (DCGM) in AD patients. In these studies, significant correlations were found between DCGM and worsening performance on measures of cognitive function (de Leon et al., 1983). Subsequent studies have revealed that DCGM in AD is not simply a global decrease in glucose use across the brain, but rather maps to specific regions found in the posterior cingulate and parietal, temporal, and prefrontal cortices. Longitudinal studies have demonstrated the progression of DCGM over the course of AD. DCGM can be observed in preclinical AD and progressively worsens as patients proceed from mild cognitive impairment (MCI) to AD (Mosconi et al., 2009).

Given the realization that AD begins decades before onset of clinical signs of dementia, it is important to investigate and develop low risk interventions that can intervene early in the course of cognitive decline. One low risk attempt to address the DCGM in AD is to induce ketosis. Ketosis is the elevation of circulating ketone bodies, namely beta-hydroxybutyrate (BHB), acetoacetate, and acetone. Ketone bodies (KB) are normally produced from the incomplete oxidation of fatty acids under conditions of low glucose availability and are readily metabolized by the brain to serve as an alternative to glucose (Owen et al., 1967). Ketosis has traditionally been induced by adherence to a ketogenic diet. Ketogenic diets were developed to mimic fasting, and have been used for decades to reduce seizure frequency in pediatric epilepsy (Freeman et al., 2006).

Ketogenic diets require strict compliance to low carbohydrate and low protein intake and long term adherence is challenging even in the best of situations. It is possible to induce ketosis without dietary modification with special fats called medium chain triglycerides (MCTs). Reger et al. (2004) studied the effects of a specific MCT called caprylic triglyceride (CT) to induce ketosis in 20 mild to moderate probable AD subjects [mean age 74.7; mean mini mental state exam (MMSE) score 22.0]. This study used a crossover design to examine the effects of acute elevation of serum KB levels on cognitive performance. A single 40 g dose of CT induced mild ketosis and a significant positive correlation between performance on the paragraph recall task and serum BHB concentration was found. In addition, significant improvement was demonstrated in the Alzheimer’s disease assessment scale-cognitive subscale (ADAS-Cog) scores in subjects who were non-carriers of *APOE4* [*APOE4*(−)] compared to those who were carriers [*APOE4*(+)].

In a follow-up study, Henderson et al. (2009) induced ketosis in mild to moderate AD subjects (mean MMSE of 23) by daily dosing of 20 g of CT for 90 days. This study was a randomized, double-blind, placebo-controlled, multicenter trial conducted at 23 clinical sites within the United States. Consistent with the earlier acute dosing study, subjects who were *APOE4*(−) demonstrated a significant change in ADAS-Cog scores compared to placebo at both days 45 and 90. As with the earlier study, post-dose serum BHB levels correlated with improvement in ADAS-Cog scores, suggesting the induction of ketosis may be beneficial to AD patients, particularly if they lack an *APOE4* allele.

## Case Report

Here, we report a case study of a 70-year-old male diagnosed with probable AD on August 31st, 2012 based on neuropsychiatric testing, MRI and FDG PET findings. The clinical manifestations of AD were predominately short term memory deficits and processing difficulties, and no affective changes or behavioral issues were present. His physical exam was non-diagnostic, and laboratory studies were all normal, including a thyroid panel and B12 level. He is a retired executive, has a generally benign past medical history, was taking no medications at the time of his presentation, and exercised and golfed regularly.

**Figure 1:**
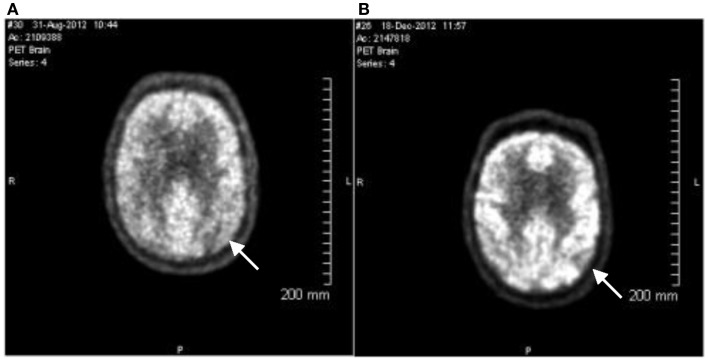
**18-Fluorodeoxyglucose (18F) positron emission tomography images**. PET images were evaluated in conjunction with CT images for both attenuation correction and anatomic fusion localization. Multiplanar PET images were obtained and evaluated as part of this study. **(A)** Image taken August 31st, 2012. Arrow indicates relative hypometabolism of right lateral parietal lobe. **(B)** Image taken December 18th, 2012. Findings are stable from the prior exam. Arrow indicates relative hypometabolism of right lateral parietal lobe. The pattern of hypometabolism involving the parietal and temporal lobes is stable and is consistent with the clinical diagnosis of probable Alzheimer’s disease.

The patient was evaluated by Montreal cognitive assessment (MoCA), MMSE, and FDG PET scans on the day of diagnosis. The MoCA is a rapid screening instrument for cognitive dysfunction. It assesses several cognitive domains and can be administered in approximately 10 min. The patient’s baseline MoCA score was 24 of a maximum 30, and the baseline MMSE was 23 of 30. In both the MoCA and MMSE, the lower the score, the more cognitively impaired the patient. The baseline FDG PET scan was done using 18.1 mCi 18F-FDG. Blood glucose during testing was 107 mg/dL, DTST 45 min. At this time, there was relatively less metabolic activity to the anterior temporal lobes and to the lateral parietal lobes, right greater than left, than to the remainder of the cortex (Figure 1A). Normal uptake was seen from the frontal and occipital lobes and basal ganglia and cerebellum. The areas of relative hypometabolism, the anterior temporal, and parietal lobes, supported the finding of probable Alzheimer type dementia. There were no findings to support frontotemporal dementia. While not conclusive, the neuropsychological testing and FDG PET images were consistent with a diagnosis of probable AD. Since previous studies had suggested that *APOE4* non-carriers responded better to induced ketosis, the patient was screened for *APOE4* carriage status and was found to be an *APOE4* non-carrier.

On the day after the Baseline evaluation, the patient was started on CT following a graduated dosing schedule. The CT formulation was provided in sachets containing a dry powder composed of 50% CT. The graduated dosing schedule began with the patient taking 10 g of material (5 g CT) for the first 2 days and then the dose was increased by 10 g (5 g of CT) every 2 days, until after 7 days, the patient was consuming the full 40 g dose (20 g CT). For the remaining 102 days, the CT formulation was administered at 40 g (20 g). The study was approved by the local Ethics Committee (High Point Regional Health System Institutional Review Board, IRB00003056). Written informed consent was obtained from the patient for the study.

**Figure 2:**
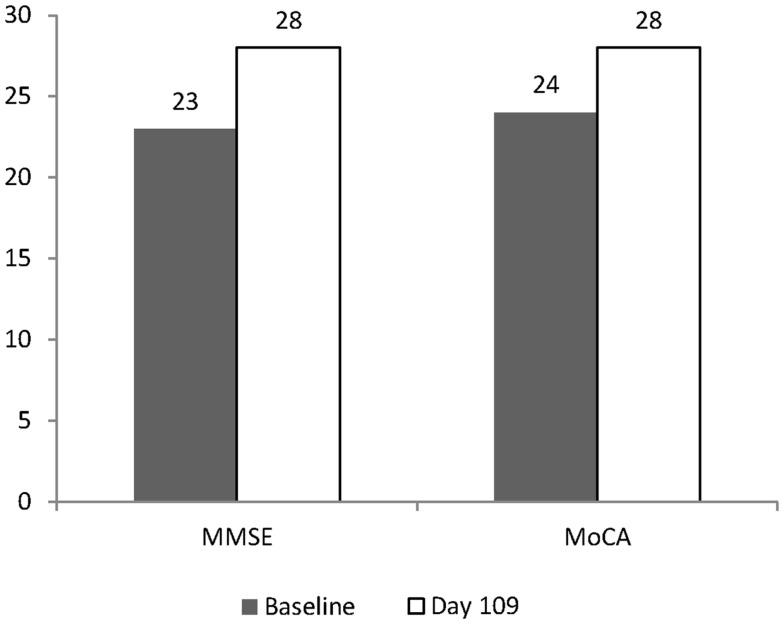
**Change in MMSE and MoCA after 109 days**. Baseline values are shown as shaded columns. Day 109 values are shown as open columns. Baseline values were obtained on August 31st, 2012. The day after the Baseline evaluation, the patient started on CT using a graduated dosing schedule for 7 days, and for the remaining 102 days was dosed at 40 g (20 g of CT). Day 109 values were obtained on December 18th, 2012.

The patient remained on 40 g/day (20 g CT) dosing for 102 days. On December 18th, 2012, the patient returned for cognitive evaluation (MoCA and MMSE) and underwent an FDG PET scan (17.5 mCi 18F-FD, blood glucose during testing was 112 mg/dL, DTST 45 min). The pattern of hypometabolism involving the parietal and temporal lobes was noted to be stable when compared to the prior exam (Figure 1B). The MoCA score changed from a baseline value of 24 to a Day 109 value of 28. The MMSE score changed from a Baseline value of 23 to a Day 109 value of 28 (Figure 2).

To evaluate the change in cognitive performance, we used a Bayesian approach to compare the scores of the patient at Baseline and at Day 109 to normative datasets of MMSE and MoCA scores using the method of Crawford and Garthwaite (2007). For the MoCA, normative scores for the patient’s age and education level of 27.4 (standard deviation, 2.2) were used (Nasreddine et al., 2005) and for MMSE, 28 (standard deviation, 1.6) were used (Crum et al., 1993) to calculate the Bayesian *p*-values for the patient’s scores, relative to the normal distribution using the program SINGLEBAYES.EXE^1^. At Baseline, the patient was significantly different from the normal score for his age and education level on the MMSE test (*p* = 0.002) but not the MoCA (*p* = 0.13). At Day 109, the patient was well within the normal range of both the MMSE (*p* = 0.99) and MoCA (*p* = 0.79) (see Table 1).

**Table 1:** **Outcomes**.

Test	Normative data	Baseline	Bayesian	Day 109	Bayesian
	Mean (SD)	Patient score	*p*-Value	Score patient	*p*-Value
MMSE	28 (1.6) (Crum et al., 1993)	23	0.002	28	0.99
MoCA	27.4 (2.2) (Nasreddine et al., 2005)	24	0.13	28	0.79

## Discussion

In this open label study, CT seems to have some ability to modulate cognitive performance as measured by the MoCA and MMSE scores. This is consistent with previous studies, which showed improvement in ADAS-Cog scores among *APOE4* non-carriers administered CT (Reger et al., 2004; Henderson et al., 2009). The ADAS-Cog test is commonly used in clinical trials testing new therapeutics for AD. However, the ADAS-Cog test is labor intensive and can take 45–60 min to administer (Rosen et al., 1984). Because of these reasons, the ADAS-Cog test is not commonly used in clinical practice. In practice, it is more practical to monitor patient outcomes with the use a rapid test such as the MoCA or the MMSE. The MoCA test is readily available and has been validated as a sensitive test of cognition in AD^2^. The present study is the first report of the use of the MoCA test with CT and suggests that the MoCA is an attractive measure of patient response.

Over the 109 day course of the study, the patient’s MoCA increased by four points and the MMSE increased by five points. This is in contrast to average changes in MMSE score reported by 12 week, double-blind studies in AD. In a meta-analysis of the effectiveness of medications for AD, little if any change in MMSE can be detected over 12 weeks (Bond et al., 2012). Since the patient described in the present study was not taking medications for AD, they can best be compared to a naïve placebo population. In studies of similar length (12 weeks), average changes in MMSE score in such populations range from 0.0 (*n* = 263) (Courtney et al., 2004) to 1.8 (*n* = 55) (Holmes et al., 2004). This is consistent with the finding that the patient was significantly different from the normal MMSE score at Baseline using the Bayesian approach. However, using this approach the MoCA score at Baseline did not yield a significant difference from the normative data set. This is likely due to the larger standard deviation in the MoCA normative data, compared to the MMSE. Yet, due to variability in the disease state and cognitive testing, the results of a single patient cannot be reliably compared to larger clinical trials.

In addition, other factors may have influenced the outcome of this study. For example, the patient was given CT as part of an open label design and hence, changes in cognitive performance may have been due to a placebo effect. Placebo effects have been documented in AD trials using placebo-controlled designs (Ito et al., 2013). Also, effects of mood, and in particular depression, have been implicated in altering scores on the MMSE test (McCall and Dunn, 2003). Therefore, the change in MMSE and MoCA scores in a case study are at best suggestive and warrant further confirmation in a larger more comprehensive study design.

The prior studies of the effects of CT in mild to moderate AD did not examine changes in cerebral glucose metabolism during the induction of ketosis. Such changes might be anticipated based on the proposed mechanism of action. Previous studies had suggested that infusion of ketone bodies would significantly alter the CMRglc metabolism. Infusion of BHB to approximately 2 mM reduces cerebral glucose metabolism by 30% (Hasselbalch et al., 1996). The present study is the first report of an attempt to measure changes in CMRglc with CT induced ketosis and may aid in the design and development of future studies. The obvious limitation of these observations is that they are derived from a single-case report.

The FDG PET images presented here indicate no observable changes in glucose uptake in any region of the brain. The level of ketone bodies produced by CT therapy is in the range of 0.4 mM (Henderson et al., 2009), much lower than the previously reported infusion levels. This would represent approximately 3–5% of total brain metabolism (Cunnane et al., 2011). Such a minor contribution to cerebral metabolism may not be detectable in the FDG PET scan reported here. This is an important observation for the design of future FDG PET studies in which mild ketosis is induced.

Alternatively, the failure to modify brain glucose metabolism may point to a different mechanism of action attributed to ketone bodies that are mediating changes in cognitive performance. For example, as well as being a fuel source, ketone bodies also act as signaling metabolites. The major ketone body BHB has been identified as an inhibitor of type I histone de-acetylases (HDAC). Inhibition of this class of HDAC by BHB has been associated with a variety of protective measures such as stress resistance, longevity, and metabolic health [for review see Newman and Verdin (2014)]. Thus, the changes in gene expression by modification of HDAC and other protein targets may be responsible for the increased MoCA and MMSE scores in the present study.

## Concluding Remarks

In conclusion, this is the first report of the use of 18FDG PET and the MoCA test in a patient administered CT for 109 days. The results suggest that CT may have an effect on cognitive function in mild AD, though glucose uptake and metabolism may remain unchanged. This finding may suggest that ketone bodies are not simply acting as an energy source, but may be working to modify gene expression through changing HDAC activity. However, due to the low level of reported ketosis and the inherent variability in the disease state and cognitive testing, the results of a single patient must be viewed in the context of a case study, and further studies are warranted.

## Conflict of Interest Statement

The author of this manuscript is a paid consultant of Accera Inc. Accera develops and has commercialized Medium Chain Triglcyeride based medical food for mild to moderate Alzhiemer’s disease.

## Footnotes

^1^http://homepages.abdn.ac.uk/j.crawford/pages/dept/BayesSingleCase.htm

^2^http://www.mocatest.org
